# Comprehensive study on in vitro propagation of some imported peach rootstocks: in vitro explant surface sterilization and bud proliferation

**DOI:** 10.1038/s41598-024-55685-3

**Published:** 2024-03-07

**Authors:** Galal I. Eliwa, El-Refaey F. El-Dengawy, Mohamed S. Gawish, Mona M. Yamany

**Affiliations:** Horticulture Department, Faculty of Agriculture, Damietta University, New Damietta, Egypt

**Keywords:** Axillary bud, Micropropagation, Proliferation, Shoot tip, Sodium hypochlorite, Biotechnology, Plant sciences, Plant reproduction

## Abstract

The present study was conducted in the Laboratory of Tissue Culture, Horticulture Department, Faculty of Agriculture, Damietta University, Egypt. The objective of this study was to establish a micropropagation protocol suitable for three imported peach rootstocks: Okinawa (*P. persica*), Nemared (*P. persica* × *P. davidiana)* × *P. persica*), and Garnem (*P. dulcis* × *P. persica*) in vitro. The results showed that soaking the explants in sodium hypochlorite (NaOCl) at 20% for 15 min produced the highest responsiveness (82.81%), survival (96.61%), with the lowest mortality (3.14%) and contamination (0.24%). Explants of the Garnem genotype had the best response (89.12%), survival (90.62%), lowest mortality (0.00%), and highest contamination (9.37%) when compared to the other genotypes. In comparison with axillary buds, the shoot tip displayed the highest responsiveness, survival, and death (100, 87.40, and 12.59%, respectively), as well as the least significant contamination (0.00%). Additionally, the percentages of responsive, survived, dead, and contaminated explants at the various collection dates varied significantly. The 6-benzylaminopurine (BAP) concentrations used (3 to 5.0 mg/L) demonstrated similar behavior in terms of in vitro proliferation, with rates of 3.77 to 6.11, 4.33 to 8.88, and 3.33 to 7.44 shoot numbers per explant for the Okinawa, Nemared, and Garnem peach rootstocks, respectively, indicating that the number of shoot proliferations is genotype-dependent. Additionally, using 5.0 mg/L BAP in combination with 0.2 mg/L IBA significantly increased average shoot proliferation (96.29%), number of shoots per explant (7.48), and average leaf number/explant (16.33) compared to the other treatments. Based on these results, adventitious bud development was enhanced during in vitro multiplication of the Okinawa, Nemared, and Garnem peach rootstocks by the synergistic interaction of indole-butyric acid (IBA) and 6-benzylaminopurine (BAP).

## Introduction

Peach (*Prunus persica* L. Batsch) is the third most important temperate tree fruit species behind apple and pear. It belongs to the subfamily *Prunoideae*, *Prunus* genus, family *Rosacea*, with 8 basic and 16 somatic chromosome numbers (2n = 16)^1^. China is the native home of peaches, which were domesticated there 4000–5000 years ago^2^. Egypt is the world’s eighth-largest peach producer behind China and the European Union, while the harvested area reached 13,757 hectares with a total production of 244,228.55 tons^3^. However, root-knot nematodes are a significant issue that can limit the expansion of peach growing in different regions of Egypt, particularly in sandy soils^4^. The management of root-knot nematodes is difficult because of their wide host range and ability to survive in diverse environmental conditions. Chemical nematicides are the most commonly used management methods; however, they are costly and contaminate the environment^5^. Hence, eco-friendly methods, such as resistant rootstocks and biological control, have attracted special attention^6^. Some *prunes* rootstocks e.g., Garnem, Okinawa, Nemaguard and Nemared, are resistant to root-knot nematodes (Meloidogyne spp.)^6,7^. Therefore, Egypt imports *prunus* rootstocks that are resistant to root-knot nematodes, such as Garnem, Nemaguard, Nemared, and Okinawa, as tissue culture seedlings or seeds via the private sector from the USA, Italy, Spain, and France each year for budded stone fruits (peaches, nectarines, plums, and almonds), which costs millions of dollars annually^4^. The use of conventional propagation methods in peaches is quite difficult because it results in a low multiplication rate^8^. *Prunus* rootstocks have a poor rooting capacity, making it challenging to propagate them on a large scale using greenwood, soft, or hardwood cuttings^4,9–12^.

Micropropagation offers a suitable method to provide growers with sufficient quantities of rootstocks pathogen-free planting material for old and new cultivars^13^. There are several processes involved in in vitro propagation, including explant selection, aseptic culture establishment, multiplication, rooting, and acclimatization of plants. One of the most important issues in micropropagation is microbial contamination^14^. Successful tissue culture of plant species depends on the removal of external and internal contaminating microorganisms^15,16^. These microorganisms include viruses, bacteria, yeast, and fungi^17^. These microorganisms compete with plant tissue cultures for nutrients. The presence of these microbes usually results in increased culture mortality but can also result in variable growth, tissue necrosis, reduction of shoot proliferation, and rooting^18,19^. Surface sterilization is the most important step in preparing explants for micropropagation because it is difficult to control fungal and bacterial contamination of woody plants from field sources^20^. Rezadost et al.^21^ found that no one sterilization method would be sufficient for all the species. Even for the same species or the same variety, a single formula may not function at different times. This is because the load and type of microorganisms on explants are dependent on the season^22^.

Sodium hypochlorite, calcium hypochlorite, ethanol, mercuric chloride, hydrogen peroxide, and silver nitrate are the commonly used disinfectants. Because these sterilizing agents are toxic to plant tissue, contamination must be removed without killing the plant cells^20,23^. Disinfectants such as ethanol and sodium hypochlorite (NaOCl) hamper the growth rate of fungi and bacteria in the growth medium^24^. It is well known that hypochlorite kills bacteria very effectively; even micromolar doses are sufficient to reduce bacterial populations significantly. Hypochlorite salts (NaOCl, CaOCl_2_) form HOCl when diluted in water, and the concentration of HOCl is correlated with bactericidal activity^25^.

Numerous elements, including genotype, culture medium, and plant growth regulators, affect micropropagation^26,27^. The most important factors in plant tissue culture, especially in the proliferation stage, are cytokinins, which play multiple roles in plant development such as the promotion of cell division, cell expansion, plant protein synthesis stimulation, and the activities of some enzymes^27–30^. However, due to genotypic differences in behavior in vitro, it is necessary to determine the ideal conditions for multiplication for each genotype. 6-benzylaminopurine (BAP) is the most effective cytokinin for in vitro multiplication of several species of the genus *Prunus*^27,31–33^. In peach tree multiplication, BAP acts in the formation and development of shoots in vitro under suitable conditions for the rooting phase^31,34–36^. The effects of cytokinins are most noticeable in tissue cultures, where they are often used together with auxins to promote cell division and regulate morphogenesis. When added to shoot culture media, these compounds defeat apical dominance and cause lateral buds to emerge from dormancy^37,38^. In addition, indole-butyric acid (IBA) could improve adventitious bud development in almonds^39^.

The micropropagation of peach rootstocks has been reported by several researches^38,40–43^. One of the biggest challenges for researchers is the development of optimal in vitro propagation protocols for the prunus species. In in vitro cultures of some peach rootstocks such as GF677 (Prunus persica x P. amygdalus), hyperhydricity and chlorosis may emerge as important problems, and shoots must be discarded as a result^27,30,44,45^. Furthermore, in Egypt, the previous studies that have been conducted on micropropagation on the current rootstocks are very limited, and it has also faced many problems during the tissue culture cycle, such as explant contamination, vetrification, and adjusting of plant growth regulators during proliferation and rooting stages. In this context, we aimed to establish a protocol for the rapid and economical micropropagation of the Garnem, Nemared, and Okinawa peach rootstocks to limit their imports from abroad and save hard currencies for our country. To fulfill this main objective, we set up the study in two consecutive phases: (i) In the first report, we laid out a procedure for explant sterilization and assessed the capacity of the Garnem, Nemared, and Okinawa peach rootstocks for in vitro multiplication under the influence of various BAP concentrations combined with IBA. (ii) In the second report, we will address the effect of IBA and NAA on the rooting and acclimatization of the Garnem, Nemared, and Okinawa peach rootstocks.

## Materials and methods

This study was conducted in the tissue culture laboratory of the Horticulture Department, Faculty of Agriculture, Damietta University, Egypt, from May 2020 to June 2023. Experimental research on plants, including collection of plant material was performed in accordance with the relevant guidelines and regulations.

### Plant material and explant preparation

Healthy and vigorous shoots about 20–25 cm long, containing 10–15 axillary buds, were collected at different dates (April, May, July, September, and October) during the growth seasons of the study from three-year-old trees of three peach rootstocks, namely, Okinawa (*P. persica*), Nemared (*P. persica* × *P. davidiana*) × *P. persica*), and Garnem (*P. dulcis* × *P. persica*), grown at the Educational Nursery of the Agriculture Faculty, Damietta University. Their leaves were cut back, leaving approximately 0.5 cm of petioles, washed under running tap water, and brought to the tissue culture laboratory.

### Explant surface sterilization

Sterilization steps were done under aseptic conditions inside the laminar airflow cabinet (Model: BBS-V1300) using sterilized instruments. Shoots were cut off into pieces 1.5–2 cm in length, each including one bud (axillary buds), while the shoot tip (meristems with two or three primordial leaves) was isolated. The explants (shoot tip and axillary buds separately) were then thoroughly washed with tap water 3–5 times, followed by liquid soap for 30 min with agitation to physically remove most microorganisms. The explants were treated with 70% ethanol for 1 min and then rinsed with distilled water three times to lower the toxic effect of ethanol. Then, they were treated with different concentrations of 10, 20, and 30% (v/v) active ingredient chlorine of locally commercial bleach sodium hypochlorite (NaOCl) with 5.25% active ingredient chlorine for 15 min to determine the exact concentration suitable for each explant type and genotype. As a wetting agent, a few drops of "Tween 80" (Sigma USA) were added to the 50-ml solution of NaOCl. After decanting the sterilizing solutions under safe conditions, the explants were washed three times each for 5 min with autoclaved distilled water to remove traces of NaOCl. Shoot tip and axillary bud explants were surface sterilized before being further trimmed to a length of about 1–1.5 cm to provide a freshly cut surface and to get rid of any sterility-damaged cells.

### Establishment stage

Surface sterilized shoot tip and axillary bud explants were cultured separately on MS medium^46^) (MS macro, microelements, vitamins) free-hormone, supplemented with 3% (w/v) sucrose as a carbon source and 3 g/L gerlite. The PH was adjusted to 5.7–5.8 by using either 1 N HCl or 1 N NaOH before the addition of gerlite. The medium was cooked and distributed into glass jars (350 mL); each jar contained about 60 mL of MS medium. Jars were finally sterilized in an autoclave for 20 min at 1.05 kg/cm^2^ and 121 °C. The establishment phase was performed in a growth room with temperature 23 ± 2 °C, with a photoperiod of 16 h of light provided by cool-white fluorescent lamps (light intensity of 2000 lx) and the relative humidity (RH) ranged from 70 to 80%. The glass jars containing cultured explants were labeled and arranged at random on the growth room shelves (Fig. 4a). Each sterilization treatment (three replicates with nine jars per replicate and three explants per jar) was lined up randomly. The experimental design was factorial with three factors (two type of explants X three concentrations (T) X three rootstocks) in a completely randomized design (CRD); each treatment consisted of three replicates with nine jars per replicate and three explants per jar. After 28 days of culturing, the sterilization experiment data recorded included the number of responsive explants, surviving (clean), and contaminated cultures (see Fig. 4b,c,d ,e. The data was converted into percentages The data was converted into percentages.

### Proliferation stage

Shoots obtained from the establishment stages at different dates of taking the explant (April, May, July, September, and October) were inoculated under aseptic conditions inside the laminar airflow cabinet (Model: BBS-V1300) in glass jars (350 mL) containing 60 mL of MS medium^46^ supplemented with 3% (w/v) sucrose, 3 g/L gerlite, 1.5 g/L activated charcoal, and different 6-benzylaminopurine (BAP) concentrations, i.e., 0, 3, 5 ppm, in combination with 0, 0.1, 0.2 ppm indole-butyric acid (IBA) (Table 1), were tested to investigate which concentration induced the highest multiplication. MS medium without plant growth regulators (PGRs) (T1) was used as a control. The plant material was kept in a growth chamber at a temperature of 23 ± 2 °C, a photoperiod of 16 h of light using cool-white fluorescent lamps (light intensity of 2000 lx), and a relative humidity (RH) of 70 to 80%. After 6 weeks, the percent of explants proliferating was calculated. The average number of shoot, shoot height, and number of leaves per explant were recorded for each treatment per rootstock. The shoot height was estimated with the aid of a caliper to evaluate all shoots per explant for each treatment and replication. The experimental design was factorial with two factors (nine treatments × three rootstocks) in a completely randomized design(CRD); each treatment consisted of three replicates with nine jars per replicate and three explants per jar.

**Table 1:** Combinations of BAP and IBA (mg/L) in multiplication media treatments (T).

BAP	0.0 ppm	3.0 ppm	5.0 ppm
IBA			
0.0 ppm	T1 (control)	T2	T3
0.1 ppm	T4	T5	T6
0.2 ppm	T7	T8	T9

### Statistical analysis

The experiments were repeated three times to confirm the results and were conducted as factorials in a completely randomized (CRD) design. Each treatment consisted of three replicates, with nine jars per replicate and three explants per jar during the establishment and proliferation phases. The results were statistically analyzed using CoStat Computer Software (version 6.311). Differences between means were evaluated using the least significant difference (LSD) test at p ≤ 0.05^47^

## Results and discussion

### Establishment stage

#### Effect of various sodium hypochlorite (NaOCl) concentrations on axillary bud explants and shoot tip surface sterilization

##### Average responsive percentage

The results are presented in Table 2 and Fig. 4b and c showed that the Garnem genotype achieved the best responsiveness (89.12%) and significantly overcame the Okinawa and Nemared genotypes with insignificant differences between them. However, the shoot tip showed the highest response (74.30%), which did not differ significantly from the axillary bud, explants (62.77%). The best response of shoot tip explants (100%) was achieved with the Garnem genotype, followed by the Okinawa genotype, which achieved (77.77%), while the least response (45.13%) was recorded with the Nemared genotype. The best response for axillary bud explants was (78.24%) with the Garnem genotype, whereas the lowest responsive (49.04%) was recorded with the Okinawa genotype.

**Table 2:** Effect of soaking the explants in various concentrations of sodium hypochlorite (NaOCl) for 15 min on the responsive percentage of Okinawa, Nemared, and Garnem rootstocks.

Rootstock (A)	Type of explant (B)	NaOCl concentration (%): 15 min. (C)			Average (AXB)
		10%	20%	30%	
Okinawa	Shoot tip	100.00a	100.00	33.33c	77.77AB
	Axillary bud	50.92bc	55.92a–c	40.27c	49.04C
Average (AXC)		75.46AB	77.96AB	36.80C	Average (A) 63.41B
Nemared	Shoot tip	33.33c	68.75a–c	33.33c	45.13C
	Axillary bud	64.58a–c	77.76a–c	40.74c	61.02BC
Average (AXC)		48.95 BC	73.25 AB	37.03 C	Average (A) 53.08B
Garnem	Shoot tip	100.00a	100.00a	100.00a	100.00A
	Axillary bud	62.50a–c	94.44ab	77.77a–c	78.24AB
Average (AXC)		81.25A	97.22A	88.88A	Average (A) 89.12A
Average (BXC)	Shoot tip	77.77ab	89.58a	55.55b	Average (B) 74.30A
	Axillary bud	59.33b	76.04ab	52.93b	Average (B) 62.77A
Average (C)		68.55AB	82.81A	54.24B	

*Means of each factor and their interaction followed by the same letters are not significantly different from each other at P ≤ 0.05 according to the LSD test.

Regarding the interaction between rootstocks, type of explants, and NaOCl concentration, the best response of shoot tip explants was (100%) achieved with the Garnem genotype at (10, 20, and 30% NaOCl) and the Okinawa genotype at 10 and 20% NaOCl, while the lowest response (33.33%) was recorded with the Nemared genotype at 10 and 30% NaOCl and the Okinawa genotype at 30% NaOCl. The best response of axillary bud explants (94.44%) was achieved with the Garnem genotype at 20% NaOCl, whereas the lowest response (40.27%) was recorded for the Okinawa genotype at 30% NaOCl. In addition, the results in Table 2 demonstrate that the use of 20% NaOCl for 15 min achieved the highest response percentage (82.81%), followed by 10%, while the use of 30% NaOCl for 15 min recorded in the lowest response percentage (54.24%).

##### Average survival percentage

Results are shown in Table 3 and Figs. 4b and c showed that the Garnem genotype achieved the best survival (90.62%), which did not differ significantly from the Nemared and Okinawa genotypes (85.19 and 83.98%, respectively). Also, shoot tip showed the highest survival (87.40%) but did not differ significantly from the axillary bud explants (85.79%). The best survival of shoot tip explants (100%) was achieved with the Garnem, followed by (84.44%), which was achieved with the Nemared genotype, while the least survived (77.77%) was recorded with the Okinawa genotype. Although the best survival for axillary bud explants (90.18%) was achieved with the Okinawa genotype, it did not differ significantly with the Nemared and Garnem genotypes (85.93 and 81.25%, respectively).

**Table 3:** Effect of soaking the explants in various concentrations of sodium hypochlorite (NaOCl) for 15 min on the survival percentage of Okinawa, Nemared, and Garnem rootstocks.

Rootstock (A)	Type of explant (B)	NaOCl concentration (%): 15 min. (C)			Average (AXB)
		10%	20%	30%	
Okinawa	Shoot tip	100.00a	100.00a	33.33c	77.77B
	Axillary bud	86.11a	94.44a	90.00a	90.18AB
Average (AXC)		93.05ABC	97.22AB	61.66D	Average (A) 83.98A
Nemared	Shoot tip	100.00a	86.66a	66.66a–c	84.44AB
	Axillary bud	83.33a	98.55a	75.92ab	85.93AB
Average (AXC)		91.66ABC	92.60A-C	71.29CD	Average (A) 85.19A
Garnem	Shoot tip	100.00a	100.00a	100.00a	100.00A
	Axillary bud	43.75bc	100.00a	100.00a	81.25AB
Average (AXC)		71.87BCD	100.00A	100.00A	Average (A) 90.62A
Average (BXC)	Shoot tip	100.00a	95.55a	66.66c	Average (B) 87.40A
	Axillary bud	71.06bc	97.66a	88.64ab	Average (B) 85.79A
Average (C)		85.53AB	96.61A	77.65B	

*Means of each factor and their interaction followed by the same letters are not significantly different from each other at P ≤ 0.05 according to the LSD test.

Regarding the interaction between rootstocks, type of explants, and NaOCl concentration, the best survival of shoot tip explants was (100%) achieved with the Garnem genotype at (10, 20, and 30% NaOCl) and Okinawa genotype at 10 and 20% NaOCl, while the least survived (33.33%) was recorded with the Nemared genotype at 30% NaOCl. The best survival of axillary bud explants (100%) was achieved with the Garnem genotype at 20 and 30% NaOCl, while the least survived (43.75%) was recorded with the Garnem genotype at 10% NaOCl. Also, the results in Table 3 demonstrate that soaking the explants in NaOCl at 20% for 15 min achieved the highest survival percentage (96.61%), followed by 10%, while using NaOCl at 30% for 15 min recorded the lowest value (77.65%).

##### Average contaminated percentage

Results are shown in Table 4 and Fig. 4d showed that the Garnem genotype recorded the maximum contamination (9.37%), while the minimum values achieved with the Okinawa and Nemared genotypes (2.87 and 4.87%, respectively) did not differ significantly. Also, the shoot tip showed the minimum significant contamination (0.00%), while the axillary bud explants recorded the maximum significant contamination (11.41%). The shoot tip explants recorded the minimum significant contamination (0.00%) with the three rootstocks. For the axillary bud explants, the maximum contaminated percentage (18.75%) was achieved with the Garnem genotype, followed by the Nemared genotype (9.74%). The minimum contaminated percentage of axillary bud explants (5.74%) was achieved with the Okinawa genotype.

**Table 4:** Effect of soaking the explants in various concentrations of sodium hypochlorite (NaOCl) for 15 min on the contaminated percentage of Okinawa, Nemared, and Garnem rootstocks.

Rootstock (A)	Type of Explant (B)	NaOCl concentration (%) 15 min. (C)			Average (AXB)
		10%	20%	30%	
Okinawa	Shoot tip	0.00e	0.00e	0.00e	0.00C
	Axillary bud	13.88bc	0.00e	3.33c–e	5.74BC
Average (AXC)		6.94BC	0.00C	1.66BC	Average (A) 2.87B
Nemared	Shoot tip	0.00e	0.00e	0.00e	0.00C
	Axillary bud	16.66b	1.45de	11.11b–d	9.74B
Average (AXC)		8.33B	0.72C	5.55BC	Average (A) 4.87B
Garnem	Shoot tip	0.00e	0.00e	0.00e	0.00C
	Axillary bud	56.25a	0.00e	0.00e	18.75A
Average (AXC)		28.12A	0.00C	0.00C	Average (A) 9.37A
Average (BXC)	Shoot tip	0.00b	0.00b	0.00b	Average (B) 0.00B
	Axillary bud	28.93a	0.48b	4.81b	Average (B) 11.41A
Average (C)		14.46A	0.24B	2.40B	

*Means of each factor and their interaction followed by the same letters are not significantly different from each other at P ≤ 0.05 according to the LSD test.

Regarding the interaction between rootstocks, type of explants, and NaOCl concentration (Table 4) the shoot tip explants recorded the minimum contaminated% (0.00%) with three genotypes at (10, 20, and 30% NaOCl).While the axillary bud explants recorded the maximum contamination (56.25%) with the Garnem genotype at 10% NaOCl. The results in Table (4) demonstrate that soaking the explants in NaOCl at 20% for 15 min achieved the minimum value of contamination (0.24%), which did not differ significantly from soaking the explants in 30% NaOCl (2.40%). Axillary bud explants show inferior results. It might be due to the aged nature of the axillary bud explants, which may be heavily contaminated as compared to the shoot tip.

From the above-mentioned results, we could explain the fact that requirements for sterilization are different and depend on the tissue type and the genotype of the explant used for micropropagation. One of the most crucial steps in the establishment stage of plant tissue culture is the sterilization process, which depends on the removal of internal and external contaminating microorganisms while lowering the plant tissue's death rate and increasing its response and survival percentages. All of these factors help for mass propagation of Okinawa, Nemared, and Garnem rootstocks in vitro. Our results are in agreement with^22,48^. The highest meristem survival (75%) was recorded in cultivars Osogrande and Toro when treated with 0.5% NaOCl for 15 min. However, 75% of the explants in Chandler survived when treated with 1% NaOCl for 10 min^49^. Similarly, maximum survival (58–71%) was observed in Chandler, Osogrande, and Islamabad Local when internodal segments were treated with 0.5% NaOCl for 15 min. However, the survival percentage of these cultivars significantly varied at various NaOCl concentrations when petiole segments were used as explants. Additionally, Sirimat and Sakulsathaporn^50^ came to the conclusion that sterilization with shoot tip and nodal explants treated with 10% NaOCl for 10 min and then 10% NaOCl for 15 min each is the most efficient method with the highest survival rates, followed by 10% NaOCl for 15 min and the nodal explants treated with 10% NaOCl for 10 min and then 5% NaOCl for 15 min. Al Ghasheem et al.^51^ worked on peach explants, and they found that sodium hypochlorite was the most effective treatment, with a 50% survival rate at 15% NaOCl for 5 min and 60% at 10% NaOCl for 10 min. The solution of sodium hypochlorite for superficial sterilization of the explant was efficient and didn't injure the explants at the appropriate concentration^52^**.** Also,^53^ and^54^ reported that the high concentration of sodium hypochlorite can be effective in sterilizing the superficial explants cultivated in vitro, but it is accompanied by the death of explants. NaOCl has widely been accepted to eliminate microorganisms since it effectively and rapidly kills vegetative spores, bacteria, fungi, protozoa, and viruses by oxidizing sulfhydryl groups of essential enzymes and proteins and damaging DNA and membranes^50^.

#### Effect of collecting dates of shoot tip and axillary bud explants and soaking them in 20% NaOCl for 15 min on their response, survival, death, and contamination percentages

**Figure 1:**
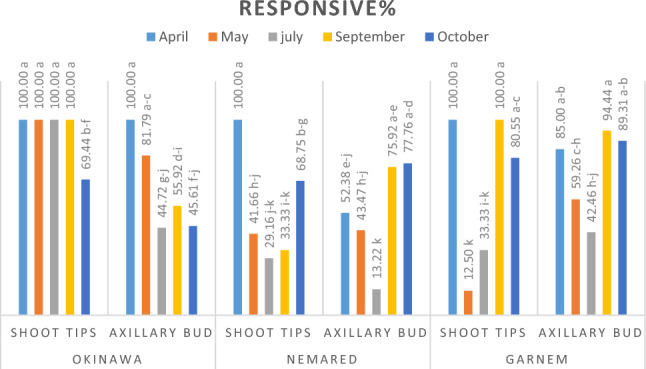
responsive% of shoot tip and axillary buds of Okinawa, Nemared, and Garnem peach rootstocks collected at different dates and soaked in 20% NaOCl for 15 min.

**Figure 2:**
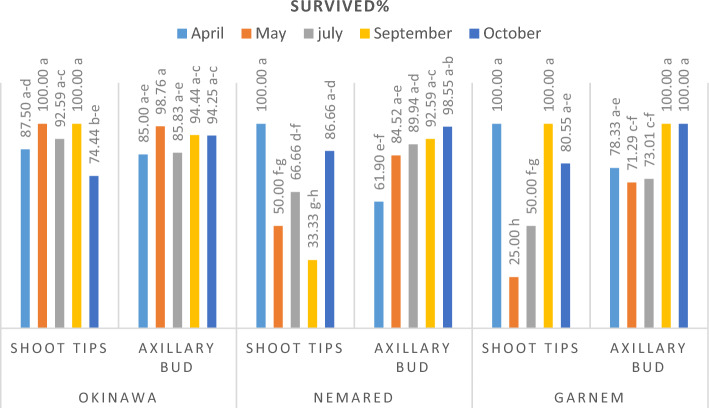
Survived% of shoot tip and axillary buds of Okinawa, Nemared, and Garnem peach rootstocks collected at different dates and soaked in 20% NaOCl for 15 min.

**Figure 3:**
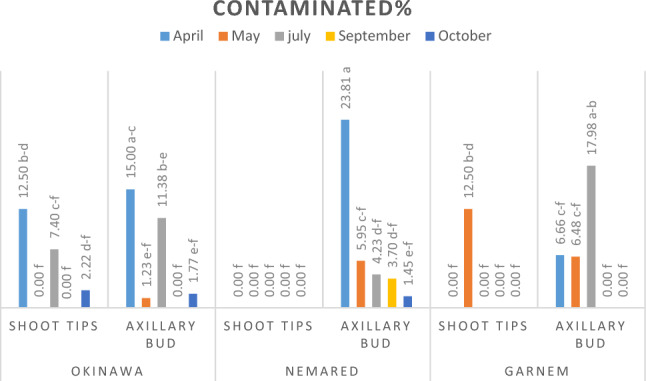
Contaminated% of shoot tip and the axillary bud of Okinawa, Nemared, and Garnem peach rootstocks collected at different dates and soaked in 20% NaOCl for 15 min.

The data illustrated in Figs. 1, 2 and 3 showed clearly that the shoot tip and the axillary bud explants differed greatly in their percentages of responsive, surviving, and contaminated at the different collected dates. Additionally, there were significant differences between the genotypes at different dates for taking the explants. Whereas shoot tips were more responsive and survived shoot regeneration and proliferation than the axillary buds at different collected dates and depended on the genotype. At various dates of collection and depending on genotype, axillary buds displayed a higher contamination percentage than shoot tip. This is because seasons have an impact on the quantity and kind of microorganisms present in explants^22^. It might be because axillary bud explants are older and more likely to be highly polluted than shoot tips. Our results are in agreement with^48–50^.

### Proliferation stage

#### Effect of BAP with or without IBA on average shoot multiplication%

**Figure 4:**
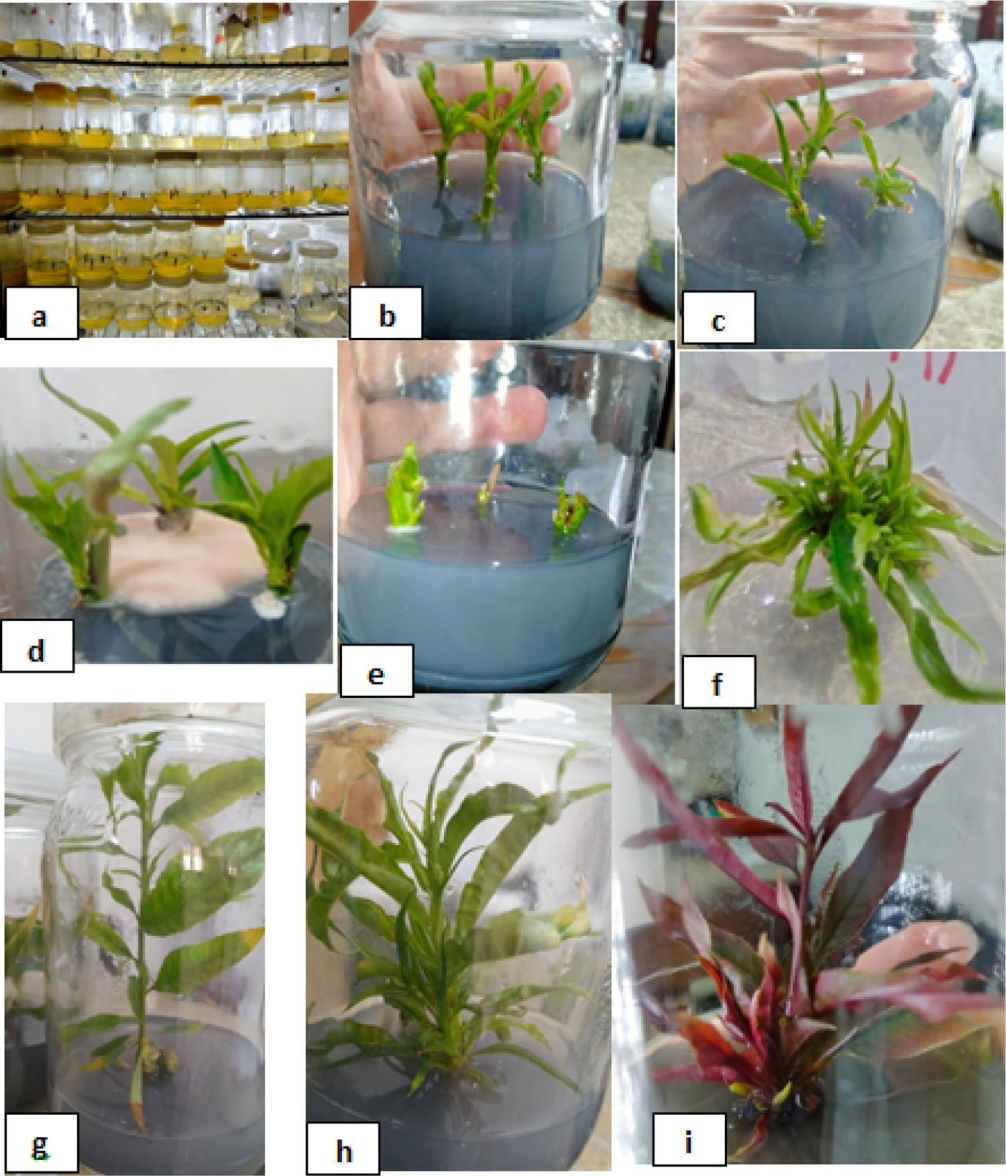
Different stages of Okinawa, Nemared, and Garnem peach rootstock micropropagation: (**a**) establishment; (**b**) soot tip responsive and survived; (**c**) axillary buds responsive and survived; (**d**) response and contamination; (**e**) no response and death; (**f**) shootlet proliferation; (**g**) shoot elongation with no proliferation; (**h**) shoot elongation with proliferation in Okinawa; and (**i**) shoot elongation with proliferation in Garnem.

The results for the average multiplication percentage of the Okinawa, Nemared, and Garnem peach rootstocks as affected by the plant growth regulators are shown in Table 5 and Fig. 4f. In general, for the different treatments, data analysis allows for the identification of two different groups. The first consisted of the treatment without the presence of 6-benzylaminopurine (BAP) in culture medium (T1, T4, and T7), where the low significant values of average shoot multiplication% were recorded (1.85, 3.70, and 9.72) in T7, T4, and T1, respectively. The second group was composed of treatments with BAP in culture medium (T2, T3, T5, T6, T8, and T9), which recorded high significant values of average shoot multiplication percentages. Also, there were clear and significant differences between the tested concentrations of BAP. The highest value was recorded with T9 (5.0BAP + 0.2IBA), which recorded (96.29%).

**Table 5:** Effect of BAP with or without IBA on the average shoot multiplication percent of Okinawa, Nemared, and Garnem peach rootstocks.

Rootstock (A)	Okinawa	Nemared	Garnem	Average (B)
Treatment (B)				
(T1)0.0BAP + 0.0IBA	0.00d	5.55cd	23.61d	9.72C
(T2)3.0BAP + 0.0IBA	88.88ab	73.61b	81.94ab	81.48B
(T3)5.0BAP + 0.0IBA	77.77b	100.00a	92.59ab	90.12AB
(T4)0.0BAP + 0.1IBA	0.00d	0.00d	5.55cd	1.85C
(T5)3.0BAP + 0.1IBA	88.88ab	94.44ab	83.33ab	88.88AB
(T6)5.0BAP + 0.1IBA	79.62ab	100.00a	92.59ab	90.74AB
(T7)0.0BAP + 0.2IBA	0.00d	5.55cd	5.55cd	3.70C
(T8)3.0BAP + 0.2IBA	94.44ab	74.07b	88.88ab	85.80AB
(T9)5.0BAP + 0.2IBA	88.88ab	100.00a	100.00a	96.29A
Average (A)	57.61A	61.47A	63.78A	

*Means of each factor and their interaction followed by the same letters are not significantly different from each other at P ≤ 0.05 according to the LSD test.

Concerning the behavior of genotype rootstock as affected by BAP and IBA combinations on average shoot multiplication%, it was clear from the data presented in Table 5 that although the Garnem genotype recorded the highest value of average shoot multiplication% (63.78%), there was no significant difference between the Nemared and Okinawa genotypes, which recorded 61.47 and 57.61%, 57.61% respectively.

Regarding the interaction between the plant growth regulators and genotypes, the data in Table 5 revealed that the maximum significant values of average shoot multiplication (100%) were recorded with high concentrations of BAP {T3 (5.0BAP + 0.0IBA mg/L), T6 (5.0BAP + 0.1IBA mg/L) and T9 (5.0BAP + 0.2IBA mg/L)} respectively, with the Nemared genotype. Also, the Garnem genotype was recorded (100%) in T9. While (T1, T4, and T7) without the presence of BAP, shoot formation did not significantly occur multiplication with the three genotypes under this study. It was clear from these results that the use of BAP is necessary for the proliferation of explants.

#### Effect of BAP with or without IBA on average number of shoots per explant

The results for the average number of shoots per explant of the Okinawa, Nemared, and Garnem peach rootstocks as affected by the plant growth regulators are shown in Table 6 and Fig. 4g, h, i. In general, for the different treatments, data analysis allows identifying two different groups. The first consisted of the treatment without the presence of 6-benzylaminopurine (BAP) (T1, T4, and T7), where shoot formation did not occur multiple. The second group was composed of treatments with BAP (T2, T3, T5, T6, T8, and T9), and the development of multiple shoots occurred. Also, there were significant differences between the tested concentrations of BAP. The highest value was recorded with T9 (5.0 BAP + 0.2 IBA mg/L), which recorded 7.48, followed by T6 (5.0 BAP + 0.1 IBA mg/L), which recorded 5.74 as the average number of shots/explant.

**Table 6:** Effect of different BAP and IBA combinations on the average number of shoots/explant of Okinawa, Nemared, and Garnem peach rootstocks.

Rootstock (A)	Okinawa	Nemared	Garnem	Average (B)
Treatment (B)				
(T1)0.0BAP + 0.0IBA	1.00i	1.00i	1.33i	1.11E
(T2)3.0BAP + 0.0IBA	3.94f–h	4.33e–h	3.33h	3.87D
(T3)5.0BAP + 0.0IBA	4.11f–h	6.22b–d	4.94d–h	5.09BC
(T4)0.0BAP + 0.1IBA	1.00i	1.00i	1.11i	1.03E
(T5)3.0BAP + 0.1IBA	3.77g-h	5.77c–f	3.33h	4.29CD
(T6)5.0BAP + 0.1IBA	4.27e–h	7.88a–b	5.05d–h	5.74B
(T7)0.0BAP + 0.2IBA	1.00i	1.11i	1.11i	1.07E
(T8)3.0BAP + 0.2IBA	5.66c–f	5.22d–g	4.77d–h	5.22BC
(T9)5.0BAP + 0.2IBA	6.11b–e	8.88a	7.44a–c	7.48A
Average (A)	3.43B	4.60A	3.60B	

*Means of each factor and their interaction followed by the same letters are not significantly different from each other at P ≤ 0.05 according to the LSD test.

Concerning the behavior of genotype rootstock as affected by BAP and IBA combinations on the average number of shoots/explant, it was clear from the data presented in Table 6 that the genotypes significantly differed in their proliferation. In this respect, the Nemared genotype produced the highest significant value (4.60) as the average number of shoots per explant. There was no significant difference between the Okinawa and Garnem genotypes, which recorded 3.43 and 3.60 as the average number of shoots per explant, respectively.

Regarding the interaction between the plant growth regulators and genotypes, the maximum significant values (8.88 and 7.88 as average number of shoots/explant) were recorded in T9 (5.0BAP + 0.2IBA mg/L) and T6 (5.0BAP + 0.1IBA mg/L), respectively, with the Nemared genotype followed by the Garnem genotype, which recorded 7.44 as average number of shoots/explant in T9. While (T1, T4, and T7), without the presence of BAP, shoot formation did not significantly occur in multiplication with the three genotypes under this study. The results in Table 6 demonstrate that the use of BAP is necessary for the proliferation of explants, but the use of IBA could improve adventitious bud development. The BAP concentrations used (3 to 5.0 mg/L) demonstrated similar behavior in terms of in vitro multiplication, with a rate of 3.77 to 6.11, 4.33 to 8.88, and 3.33 to 7.44 shoots per explant for the Okinawa, Nemared, and Garnem peach rootstocks, respectively, indicating that the number of shoots is genotype-dependent. These results are in agreement with previous reports by^34^ for five Prunus rootstocks in concentrations of 0.5 and 0.7 mg/L BAP. However, for three Prunus rootstocks, ^35^ obtained higher rates of in vitro multiplication, with values ranging from 10.5 to 16.0 shoots, indicating that the number of shoots is genotype-dependent^31^, working with the *prunus* rootstocks, obtained the highest number of shoots (25.9) in response to BAP concentration at 1.5 mg/L^55^, working on Tetra (*Prunus* empyrean 3) rootstock, found that the highest number of shoots per explant (30.4) was on ME (media created specifically) medium supplemented with 0.8 mg/L BAP and 0.05 mg/L IBA. While^42^ were working on Garnem rootstock, they indicated that the maximum mean number of shoots (7.3–7.7) per explant was found on MS medium containing 2 mg/L BAP alone and in the combination of 2 mg/L BAP, 0.5 mg/L GA3, and 0.01 mg/L IBA. Also, they reported that an increase in the concentration of BAP beyond the optimal level reduced the number of shoots, indicating an upper limit in concentration. The effect of cytokinins is most noticeable in tissue cultures, where they are often used together with auxins to promote cell division and regulate morphogenesis. These compounds, when added to shoot culture media, defeat apical dominance and cause lateral buds to emerge from dormancy^37,38^.

#### Effect of BAP with or without IBA on average shoots length (cm)/explant

The effect of treatments was clear from Table 7. The longest average length of shoots (3.05 cm) was achieved from the explants cultured on medium T1 in the control treatment (absence of BAP), whereas the shortest average shoot length/explant (2.47 cm) was achieved with T2 (3.0 BAP + 0.0 IBA). There were no significant differences between the other treatments.

**Table 7:** Effect of different BAP and IBA combinations on average shoots length (cm)/explant of Okinawa, Nemared, and Garnem peach rootstocks.

Rootstock (A)	Okinawa	Nemared	Garnem	Average (B)
Treatment (B)				
(T1)0.0BAP + 0.0IBA	2.90a–c	2.93a–c	3.31a	3.05A
(T2)3.0BAP + 0.0IBA	2.96a–c	2.45a–c	2.01c	2.47B
(T3)5.0BAP + 0.0IBA	2.63a–c	2.54a–c	2.72a–c	2.63AB
(T4)0.0BAP + 0.1IBA	3.22a	2.26b–c	3.00a–b	2.82AB
(T5)3.0BAP + 0.1IBA	2.74a–c	2.66a–c	2.36a–c	2.59AB
(T6)5.0BAP + 0.1IBA	2.76a–c	2.17b–c	2.57a–c	2.50AB
(T7)0.0BAP + 0.2IBA	3.31a	2.77a–c	2.87a–c	2.98AB
(T8)3.0BAP + 0.2IBA	2.70a–c	2.81a–c	3.26a	2.92AB
(T9)5.0BAP + 0.2IBA	2.74a–c	2.47a–c	3.24a	2.82AB
Average (A)	2.88A	2.56B	2.81AB	

*Means of each factor and their interaction followed by the same letters are not significantly different from each other at P ≤ 0.05 according to the LSD test.

Concerning the behavior of genotype rootstock as affected by BAP and IBA combinations, it was clear from the data presented in Table 7 that the three rootstocks significantly differed in shoot length as affected by the treatments. The Okinawa genotype produced the longest shoot length (2.88 cm), which was followed by the Garnem genotype (2.81 cm), while the shortest value was recorded with the Nemared (2.56 cm).

Regarding the interaction between the plant growth regulators and genotypes, there was no clear trend for the behavior of the three genotypes with the treatments in this respect. The Okinawa genotype recorded high values (3.31 and 3.22 cm) in T7 and T4, respectively. While the high values with the Garnem genotype were recorded with T1, T8, and T9 (3.31, 3.26, and 3.24 cm, respectively), there were no significant differences between the treatments with the Nemared genotype (Table 7). These results are in agreement with those observed for the peach tree^35^ and plum tree^29,32^. The superiority in the length of the shoots in the media that contained low concentrations of BAP (0.00 mg/L), compared to the other treatments could be attributed to the decrease in the number of shoots in these treatments, and thus the opportunity for them to obtain the nutrient from the medium increased compared to the treatments that contained high concentrations (3 and 5 mg/L) of BAP, which formed more shoots^56–58^.

#### Effect of BAP with or without IBA on average leaf number/explant

Concerning the behavior of genotype rootstock as affected by BAP and IBA combinations on average leaf number/explant, it was clear from the data presented in Table 8 that, although the highest significant average leaf number/explant (16.12) was achieved by the Okinawa genotype, it did not significantly differ from the Garnem genotype (13.97). While the lowest average leaf number/explant (9.87) was recorded with the Nemared genotype.

**Table 8:** Effect of different BAP and IBA combinations on average leaf number/explant of Okinawa, Nemared, and Garnem peach rootstocks.

Rootstock (A)	Okinawa	Nemared	Garnem	Average (B)
Treatment (B)				
(T1)0.0BAP + 0.0IBA	15.44b–g	11.77b–h	13.66b–h	13.62AB
(T2)3.0BAP + 0.0IBA	18.33a–c	7.55h	9.22f–h	11.70B
(T3)5.0BAP + 0.0IBA	12.88b–h	7.77h	11.83b–h	10.83B
(T4)0.0BAP + 0.1IBA	11.66c–h	12.00b–h	12.11b–h	11.92B
(T5)3.0BAP + 0.1IBA	17.83a–d	11.00d–h	10.77d–h	13.20AB
(T6)5.0BAP + 0.1IBA	16.61a–e	9.44e–h	14.11b–h	13.38AB
(T7)0.0BAP + 0.2IBA	17.44a–d	11.22c–h	11.83b–h	13.50AB
(T8)3.0BAP + 0.2IBA	19.00a–b	8.44g–h	17.11a–d	14.85AB
(T9)5.0BAP + 0.2IBA	15.88b–f	9.66e–h	23.44a	16.33A
Average (A)	16.12A	9.87B	13.97A	

*Means of each factor and their interaction followed by the same letters are not significantly different from each other at P ≤ 0.05 according to the LSD test.

For the effect of BAP with or without IBA treatments, it was clear from Table 8 that the highest average leaf number/explant (16.33) was achieved from the nodes cultured on medium T9 (5.0 BAP + 0.2 IBA mg/L), followed by (14.85) with T8 (3.0 BAP + 0.2 IBA mg/L), whereas the lowest average leaf number/explant (10.83) was achieved with T4 (0.0 BAP + 0.1 IBA mg/L).

Regarding the interaction between the plant growth regulators and genotypes, the data in Table 8 also revealed that the highest significant value (23.44) as average leaf number/explant was obtained in T9 (5.0 BAP + 0.2 IBA mg/L) with the Garnem genotype, which differed significantly from all other interactions. The lowest average leaf number/explant (7.55) was achieved in T2 (3.0 BAP + 0.0 IBA mg/L) with the Nemared genotype.

According to the abovementioned findings, cytokinins may play a role in reducing the effectiveness of apical dominance and their role in the vascular differentiation of lateral buds, which facilitates the growth and branching of these buds, as well as their positive effect on BAP in producing the best response during the proliferation stage compared to the control treatment (without adding BAP). Additionally, it plays a role in attracting and accumulating metabolites at the sites of lateral buds, promoting the synthesis of RNA, protein, and chlorophyll, and stimulating the growth of lateral buds^59^. BAP has been employed in the in vitro multiplication of *Prunus* rootstock^34–36^. However, a high concentration of this growth regulator can induce a reduction in bud elongation and hyperhydricity in *Prunus spp*^29,31,32^.

## Conclusion

This study presents an effective protocol for surface sterilization and proliferation of shoot tip and axillary bud explants of the peach rootstocks “Okinawa, Nemared and Garnem” in vitro micropropagation, with promising results for large-scale propagation. The most promising sterilization procedures for micropropagation are conducted with 70% ethanol and 20% sodium hypochlorite (NaOCl) for 15 min to obtain the best response, survival, and less death and contamination of the explants. The results demonstrate that the use of BAP is necessary for the proliferation of explants, but the use of IBA could improve adventitious bud development. Using 5.0 mg/L BAP in combination with 0.2 mg/L IBA significantly increased average shoot proliferation% as well as number of shoots per explant and average leaf number/explant compared to the other treatments for the Okinawa, Nemared and Garnem peach rootstocks in vitro micropropagation. Furthermore, further research is necessary to advance the peach rootstock micropropagation technique to a mass production level that promotes development and yields promising rootstocks that are appropriate for all cultivation conditions.

## Data Availability

The authors declare that all relevant data are included in the article.
